# Anthropology at the Universities

**Published:** 1896-02-01

**Authors:** A. Macalister


### Anthropology at the Universities.

By Professor A. Macalister, F.R.S.

May I call your attention to the remarks in your number (December 21st, 1895, page 195) on "Anthropological Study in Cambridge"? Anthropology is recognised as an integral part of human anatomy, and thus forms one of the subjects of examination for the Natural Sciences Tripos, and an avenue to a B.A. degree. Human anatomy in the Tripos examination has for years been recognised as consisting of two parts—(1) human morphology, and (2) anthropology—and the papers are set in these subjects.

For several years I used to lecture on anthropology in the Lent Term, and give demonstrations, but as this was found insufficient, I have arranged with a distinguished traveller and anthropologist, Professor A. C. Haddon, M.A., of Christ's College, to give lectures and practical instruction during Lent and Easter Terms each year. Last year seventy men answered the anthropological questions at the Natural Sciences Tripos examination. I may also add that at least one Fellowship has been filled up by a candidate whose distinction was chiefly his knowledge of anthropology.

Professor Ridgeway treats of the archæological side of anthropology in his lectures, and Baron von Hügel, the curator of the Museum of General and Local Archæology, gives museum demonstrations on the anthropological material under his charge, which, if not as extensive or as well housed as the Pitt-Rivers collection, is far from being "fragmentary" or "miserably stocked in sheds." The collection is enormously overcrowded, and we hope to see it some day in a much larger space, where it can be better displayed. The splendid collection of crania of different races, containing over 2,300 specimens, is well housed and displayed in the Anatomical Museum, and I may add that during last year five students in this department published monographs on anthropological subjects from work done here.

---

A Reply. By Our Contributor.

We regret very much that we should have conveyed a false impression of our meaning, and take this opportunity of expressing it more fully.

We should note, to begin with, that Professor Macalister's statements refer simply to the study of the races and physical characteristics of mankind; which, of course, is quite in its place as a department of human anatomy. The human anatomy papers referred to, however, could not be answered adequately without very detailed study of human morphology, which occupies far the larger place, so that even in this limited sense a student could not hardly be said to get the B.A. on "anthropology."

This is, however, only one section of what we have throughout taken "anthropology" to mean; and Professor Macalister has himself presided over an "anthropological" institute which is very far from confining itself to physical anthropology.

The object of our criticism was to insist upon these four points: (1) That museum accommodation at Cambridge is inadequate; which Professor Macalister admits. The collection of crania is extremely good, and well housed in the Anatomical Museum; but is quite disconnected from the anthropological collections under Baron von Hügel, which correspond with the Pitt-Rivers at Oxford, and to which our remarks were intended to apply. An impression of fragmentariness is certainly conveyed by the fact that on repeated visits we found only three sections represented in the show cases, and these in very great detail—local archæology, stone and bronze implements (the Porter bequest), and Polynesian anthropology. (2) It is true that on Professor Macalister's showing we under-estimated the number of students; but the questions answered are all on strictly physical anthropology. Moreover, as a sub-division of human anatomy "anthropology" practically comes within the reach of none but medical students; and the medical students who come into administrative or any other contact with native races abroad—which is what we were discussing—are not numerous. As we said, the students who exist are "mostly not of the kind which turns its learning to practical account." (3) We contended—whether justly or not—that there ought to be a definite course of training in anthropology in the widest sense; but that at neither University is it possible for a student to devote himself thus to the "Study of Man" and yet emerge with a degree. Oxford last year definitely repudiated—though from very mixed and mainly irrelevant motives—the proposal to establish such a "final school" of anthropology. At Cambridge the question does not appear to have arisen at all. Physical anthropology, as Professor Macalister says, is one subject in the human anatomy examination of the Natural Sciences Tripos, and that is all. The only lectures announced under this head are those of Professor Haddon—a two-terms course, once a week. Those of Baron von Hügel are not announced in the "University Reporter." Professor Ridgeway is announced, under "Art and Archæology," to lecture on "Greek and Roman Coins and Gems," and on "Mythology in Art" (*i.e.*, Greek vases). Professor Haddon, however, does supply—to those who look for anthropological lectures under "Art and Archæology"—a solid grounding in the study of the beginnings and growth of civilisation in his two-terms course on "The Elements of Sociology." (4) This last is the kind of instruction which we indicated as most desirable, as a corrective to the very narrow and specialised view generally taken by teachers of classical and mediæval, and to some extent also of modern history, and culture. But it is instructive and characteristic that anthropological instruction comes under no head of its own, but must be looked for either under human anatomy, or, so far as sociology is concerned, under "Art and Archæology."

The proposal which was rejected at Oxford was practically to elevate anthropology, which now stands, like mineralogy, as a "special subject" which may be, but never is, taken as an appendage to morphology, chemistry, or any other final school—into a separate final school, with a syllabus modelled on the very comprehensive and well-proportioned one of the existing "special subject." At present, all that is provided is an elementary course of physical anthropology by the Professor of Human Anatomy, a three-terms elementary course on the beginnings of political, social, and industrial life, by the Professor of Anthropology, occasional advanced courses by the Curator of the Pitt-Rivers Museum, and still rarer excursions into Mediterranean anthropology in connection with "Classical Art and Archæology." All special and advanced teaching is a matter of private arrangement.

